# Mutual Information as a Performance Measure for Binary Predictors Characterized by Both ROC Curve and PROC Curve Analysis

**DOI:** 10.3390/e22090938

**Published:** 2020-08-26

**Authors:** Gareth Hughes, Jennifer Kopetzky, Neil McRoberts

**Affiliations:** 1 SRUC (Scotland’s Rural College), The King’s Buildings, Edinburgh EH9 3JG, UK; 2 Department of Plant Pathology, University of California, Davis, CA 95616, USA; jlkopetzky@ucdavis.edu (J.K.); nmcroberts@ucdavis.edu (N.M.)

**Keywords:** diagnostic test, mutual information, prevalence, PROC curve, positive predictive value, negative predictive value, ROC curve, sensitivity, specificity

## Abstract

The predictive receiver operating characteristic (PROC) curve differs from the more well-known receiver operating characteristic (ROC) curve in that it provides a basis for the evaluation of binary diagnostic tests using metrics defined conditionally on the outcome of the test rather than metrics defined conditionally on the actual disease status. Application of PROC curve analysis may be hindered by the complex graphical patterns that are sometimes generated. Here we present an information theoretic analysis that allows concurrent evaluation of PROC curves and ROC curves together in a simple graphical format. The analysis is based on the observation that mutual information may be viewed both as a function of ROC curve summary statistics (sensitivity and specificity) and prevalence, and as a function of predictive values and prevalence. Mutual information calculated from a 2 × 2 prediction-realization table for a specified risk score threshold on an ROC curve is the same as the mutual information calculated at the same risk score threshold on a corresponding PROC curve. Thus, for a given value of prevalence, the risk score threshold that maximizes mutual information is the same on both the ROC curve and the corresponding PROC curve. Phytopathologists and clinicians who have previously relied solely on ROC curve summary statistics when formulating risk thresholds for application in practical agricultural or clinical decision-making contexts are thus presented with a methodology that brings predictive values within the scope of that formulation.

## 1. Introduction

Receiver operating characteristic (ROC) curves and predictive receiver operating characteristic (PROC) curves are graphical formats with application in the determination of threshold values for proxy variables used in disease risk assessment when it is, for whatever reason, deemed inappropriate to use the gold standard. The work described in the present article concerns graphical threshold determination for binary predictors based on 2 × 2 prediction-realization tables. In crop protection decision making, binary tests are disease predictors that provide a probabilistic risk assessment of, for example, epidemic vs. no epidemic, or treatment required vs. no treatment required. Context for the work described here is provided by four previous articles; in chronological order of publication, Vermont et al. [1], Shiu and Gatsonis [2], Reibnegger and Schrabmair [3] and Hughes [4]. Vermont et al. [1] described general strategies of threshold determination for both ROC curves and PROC curves. Shiu and Gatsonis [2] described PROC curves and discussed a probabilistic measure of performance. Reibnegger and Schrabmair [3] described ROC curves and discussed both probabilistic and information theoretic measures of performance. Hughes [4] described both ROC curves and PROC curves and briefly discussed both probabilistic and information theoretic measures of performance for the latter.

Both ROC curves and PROC curves are based on graphical plots of conditional probabilities. In the case of the more well-known ROC curve, the probabilities are conditioned on the actual (gold standard) disease status. For the PROC curve, the probabilities are conditioned on the outcome of the test. The shape of an ROC curve is independent of disease prevalence, whereas the shape of a PROC curve varies with prevalence. Performance measures for both ROC and PROC curves are metrics that are deployed to search for a suitable balance of the conditional probabilities on which the plots are based. Much more work has been done on describing performance measures for ROC curves than for PROC curves, reflecting the historical levels of application of the curves in the evaluation of disease predictors. The work discussed here is presented as a unifying approach to the description of performance measures for both types of curve.

To illustrate this approach, we first extend the scope of [3], a study of performance measures for ROC curves, by calculating the corresponding PROC curves. This then provides a context for a discussion of performance measures as characterized in [2,3,4] in a range of ROC curves and the corresponding PROC curves. In particular we investigate the properties of the information theoretic performance measure mutual information, applied to both ROC curves and PROC curves. The work of Vermont et al. [1] is of interest in that although it appears to be one of the earliest studies of the application of both ROC and PROC curves to the problem of probabilistic risk assessment, it has not always been cited in the subsequent literature. Thus, we will integrate a discussion of [1] with our analysis of the findings of the present study.

The methodology described here is applicable to the development of binary prediction tools in phytopathology and also in clinical medicine. In particular, we show that the adoption of an information theoretic approach to performance measurement allows the choice of an appropriate risk score threshold to take both ROC curve and PROC curve characteristics into account in a single analysis.

## 2. Methods

### 2.1. Background to ROC Curves and PROC Curves

The present analysis of ROC curves and PROC curves uses the same starting point as a previous study of some performance measures for ROC curves [3]. However, it is helpful at the outset to place the analysis in the context of the kind of phytopathological studies in which these graphical formats find application for the evaluation of disease predictors in practice.

In crop protection decision making, an ROC curve is based on the analysis of a data set that typically comprises two observations derived from agronomic data collected during the growing season from each of a set of experimental crops, untreated for the disease in question. One observation is the gold standard disease assessment, often a measure of disease intensity, yield, or quality, made at the end of the growing season. The other observation is a risk score, based on data collected earlier in the season. The risk score provides a basis for crop protection decision making because in practice, a gold standard observation would come too late for application in decision making. Risk scores are typically calibrated so that higher scores are indicative of greater probability of a disease outbreak, or of the need for a disease management intervention. The methods we describe here assume that this data set of gold standard observations and their corresponding risk scores is already available for analysis. For further information on the assembly of such a data set, see Hughes [5] for background on methods for the calculation of risk scores from agronomic data, and Yuen et al. [6] and Twengström et al. [7] for an example of the experimentation that underlies the necessary agronomic data collection.

Crops are classified as cases (‘*c*’) or non-cases (‘*nc*’), based, respectively, on whether or not the gold standard end-of-season assessment is indicative of economically significant damage. We may then calculate histograms of risk scores separately for the *c* and *nc* crop categories. Now, consider the introduction of a threshold on the risk score scale. Scores above the threshold are designated ‘+’, indicative of (predicted) need for a crop protection intervention. Scores at or below the threshold are designated ‘−’, indicative of (predicted) no need for a crop protection intervention.

The proportion of + predictions made for *c* crops is referred to as the true positive proportion (TPP or sensitivity) written *p*_+|*c*_ in conditional probability notation. The complementary false negative proportion (FNP) is written *p*_−|*c*_. Similarly, the proportion of + predictions made for *nc* crops is referred to as the false positive proportion (FPP), written *p*_+|*nc*_. The complementary true negative proportion (TNP or specificity) is written *p*_−|*nc*_. Thus, sensitivity and specificity are metrics defined conditionally on actual disease status. The ROC curve, which has become a familiar device in crop protection decision support following the pioneering work of Jonathan Yuen and colleagues [6,7], is a graphical plot of probabilities *p*_+|*c*_ (sensitivity) against *p*_+|*nc*_ (1 − specificity) derived by systematically varying the position of the threshold on the risk score scale and plotting the resulting probabilities over a range of risk scores.

In practice, the application of this analysis depends on the adoption of a particular threshold risk score for use in a given crop protection context. The variable that characterizes the risk score together with the adopted threshold on the risk score scale characterize a classification rule that may be referred to as a (binary) test (‘predictor’ is synonymous). Since the values of sensitivity and specificity are linked, a disease predictor based on a particular threshold must represent values chosen in order to achieve an appropriate balance; see Madden [8] for discussion. The considerations underlying adoption of a particular threshold risk score for use in a given crop protection context are beyond the scope of this article.

While sensitivity and specificity are of interest in characterizing a test, they are of limited significance in terms of the way we consider test results in the context of crop protection decision making. This is because they are metrics conditioned on the actual disease status which, in a practical decision-making context, we do not know. If we begin with a disease prevalence denoted *p_c_*, often what we would really like to know is the predicted probability after a + test result, denoted *p_c_*_|+_. To obtain this and similar probabilities, we apply Bayes’ Rule.

Generally, we can write *i* = +, − (for the predictions) and *j* = *c*, *nc* (for the realizations). The *p_i_* for a prediction either of intervention required (*i* = +) or of intervention not required (*i* = −) can be written as *p_i_* = *p_i_*_|*c*_∙*p_c_* + *p_i_*_|*nc*_∙*p_nc_* from the Law of Total Probability. The *p_j_* for case (*j* = *c*, prevalence) or non-case (*j* = *nc*) status, such that *p_nc_* = 1 − *p_c_*, are taken as Bayesian prior probabilities (i.e., before the test is used to make a prediction). From Bayes’ Rule, *p_i|j_*∙*p_j_* = *p_j_*_|*i*_∙*p_i_*, so we have *p_c_*_|+_ = (*p*_+|*c*_∙*p_c_*)/*p*_+_ (positive predictive value, PPV) and the complement *p_nc_*_|+_ = 1 − *p_c_*_|+_. Here, PPV refers to correct predictions of the need for a crop protection intervention; the complement 1 − PPV refers to incorrect predictions of the need for an intervention. We also have *p_nc_*_|−_ = (*p*_−|*nc*_∙*p_nc_*)/*p*_−_ (negative predictive value, NPV) and the complement *p_c_*_|−_ = 1 − *p_nc_*_|−_. Here, NPV refers to correct predictions of no need for an intervention; the complement 1 − NPV refers to incorrect predictions of no need for an intervention. The predictive values are Bayesian posterior probabilities, calculated after obtaining the prediction. We note that the positive and negative predictive values are metrics conditioned on the test outcomes. Also, unlike sensitivity and specificity, which are independent of disease prevalence, the positive and negative predictive values vary with prevalence. The PROC curve is a graphical plot of probabilities *p_c_*_|+_ (PPV) against *p_c_*_|−_ (1 − NPV).

### 2.2. Analytical Scenarios and the Calculation of ROC Curves and Corresponding PROC Curves

Reibnegger and Schrabmair [3] described four scenarios “with quite different distributional characteristics”. Each scenario comprised a pair of statistical probability distributions, modelling the separate (normalized) histograms of risk scores for *c* and *nc* subject categories. Here, we begin with the same four scenarios (Table 1).

**Figure 1:**
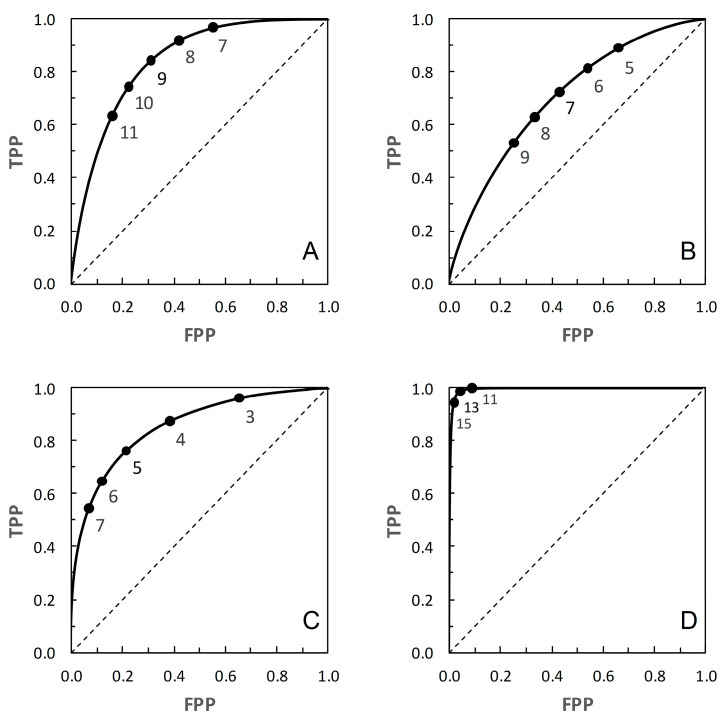
ROC curves for: (**A**) Scenario 1. (**B**) Scenario 2. (**C**) Scenario 3. (**D**) Scenario 4. See Table 1 for details. Risk score thresholds are calibrated in units of 1 unit on a 1 to 30 scale, following [3]. The risk score threshold increases along the curve from the top right-hand corner to the bottom left-hand corner. On each curve a subset of thresholds is indicated.

In Table 1, each scenario’s pair of distributions implicitly describes a parametric ROC curve. However, Reibnegger and Schrabmair [3] did not make these ROC curves explicit. Instead they used each pair of distributions as the basis for sampling *c* and *nc* data sets of various sizes. Their simulation study of ROC curve performance measures was based on the resulting sample data. Understandably, then, Reibnegger and Schrabmair [3] had no need to discuss the underlying parametric ROC curves and their properties. Here, however, these curves provide a basis for further analysis, so we explicitly calculate the ROC curve for each scenario (Figure 1) and discern its properties. An important reason for using the parametric ROC curves, rather than adopting the simulation approach of [3], is that we wish to be able to discuss the shape properties of the ROC and corresponding PROC curves for each scenario. The parametric ROC curves provide us with a non-varying baseline for this purpose. Visually, the curve for Scenario 4 passes noticeably closer to the top left-hand corner of the plot than the others, the curve for Scenario 2 stays noticeable further from the top left-hand corner, while the curves for Scenarios 1 and 3 are intermediate (Figure 1). By visual inspection, none of these ROC curves appears markedly asymmetrical.

ROC curves are often described in terms of being “proper” or “improper”. A proper ROC curve has a negative second derivative (i.e., decreasing slope) over the whole range; such a proper ROC curve never crosses the main diagonal of the plot [11]. However, an ROC curve that does not cross the diagonal may still be improper [11]. From the literature, Scenario 2 provides a proper ROC curve [9], and it appears from [10] that Scenario 1 provides an improper curve. We found no information relating to the curves for Scenarios 3 and 4. For the purpose of the present study, it is of more interest whether or not an ROC curve crosses the diagonal than whether it is strictly defined as proper or improper, so all we can really draw for certain from the literature is that the ROC curve in Figure 1B does not cross the main diagonal.

**Figure 2:**
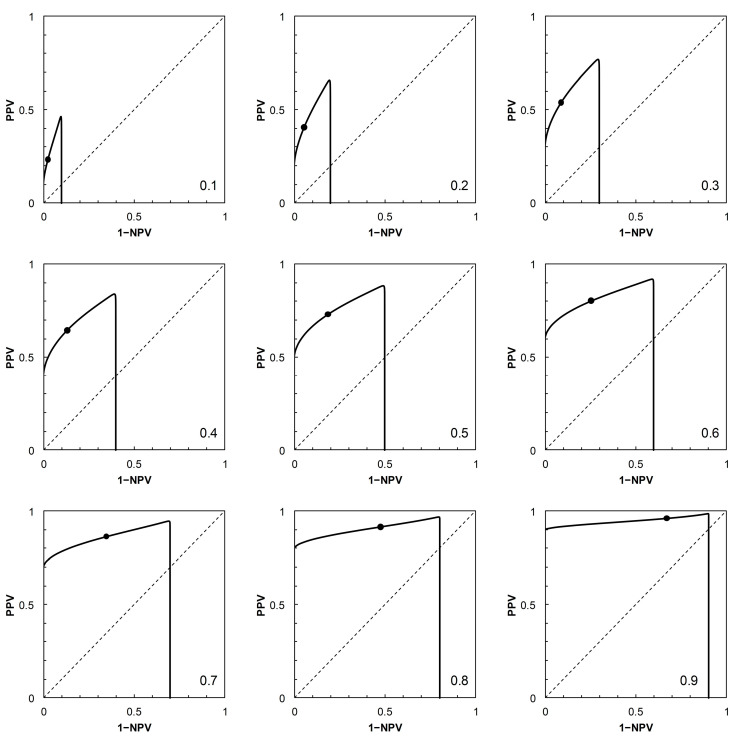
Scenario 1: Predictive receiver operating characteristic (PROC) curves corresponding to the ROC curve in Figure 1A. Each panel is labelled with the prevalence value at which the graph was calculated. For reference to Figure 1A, the threshold risk score at 9 is marked on each graph. Threshold risk scores increase along the curves, starting from the vertical axis (where 1 − NPV = 0), crossing the main diagonal (at which point PPV = 1 − NPV = prevalence) from above, and continuing the horizontal axis (where PPV = 0). NPV: negative predictive value, PPV: positive predictive value.

**Figure 3:**
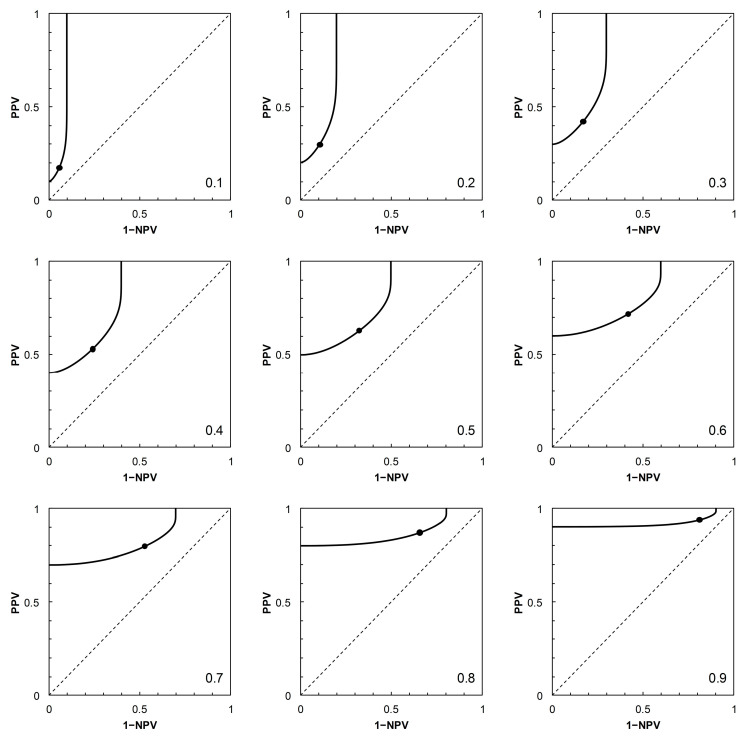
Scenario 2: PROC curves corresponding to the ROC curve in Figure 1B. Each panel is labelled with the prevalence value at which the graph was calculated. For reference to Figure 1B, the threshold risk score at 7 is marked on each graph. Threshold risk scores increase along the curves, starting from the vertical axis (where 1 − NPV = 0) and continuing to the upper horizontal of the plot (where PPV = 1) without crossing the main diagonal.

**Figure 4:**
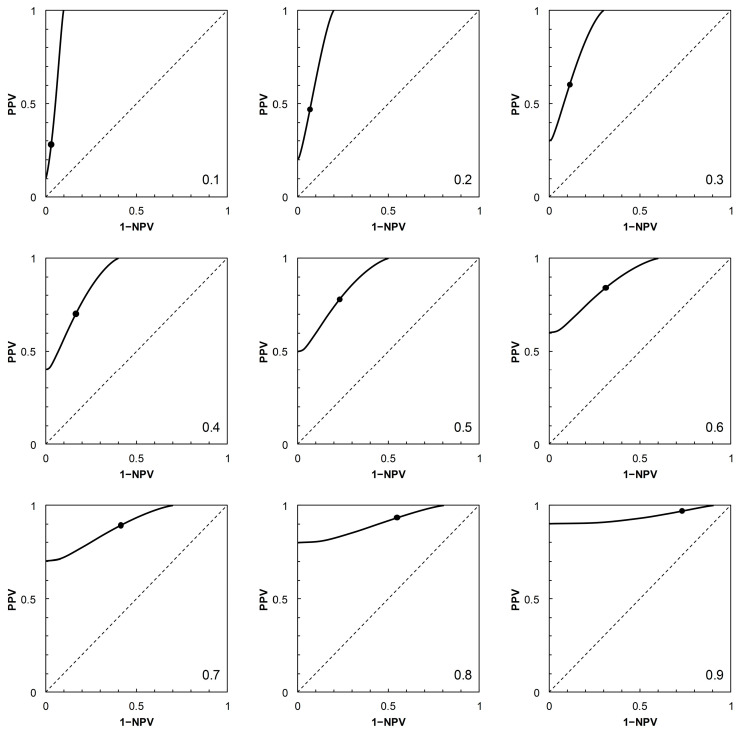
Scenario 3: PROC curves corresponding to the ROC curve in Figure 1C. Each panel is labelled with the prevalence value at which the graph was calculated. For reference to Figure 1C, the threshold risk score at 5 is marked on each graph. Threshold risk scores increase along the curves, starting from the vertical axis (where 1 − NPV = 0) and continuing to the upper horizontal of the plot (where PPV = 1) without crossing the main diagonal.

**Figure 5:**
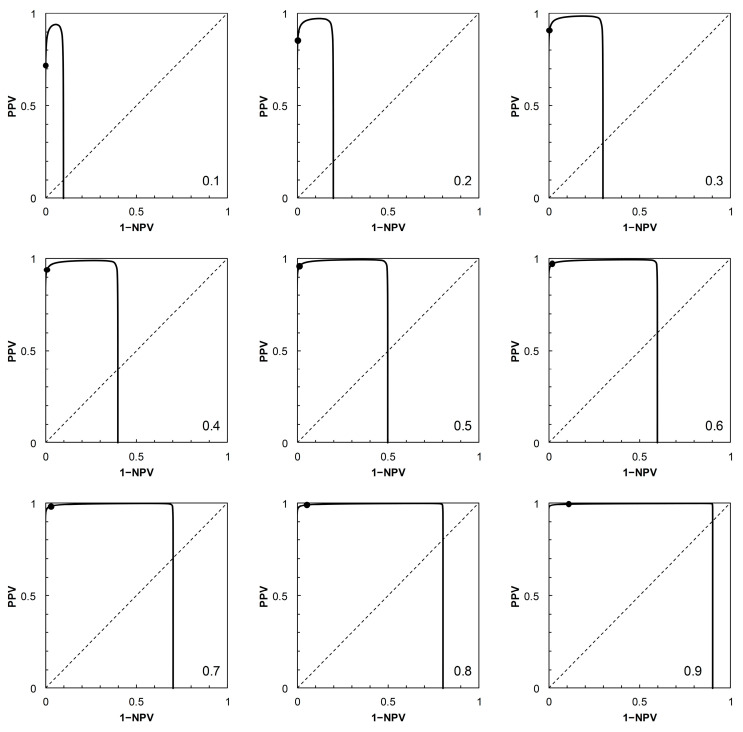
Scenario 4: PROC curves corresponding to the ROC curve in Figure 1D. Each panel is labelled with the prevalence value at which the graph was calculated. For reference to Figure 1D, the threshold risk score at 13 is marked on each graph. Threshold risk scores increase along the curves, starting from the vertical axis (where 1 − NPV = 0), crossing the main diagonal (at which point PPV = 1 − NPV = prevalence) from above, and continuing to the horizontal axis (where PPV = 0).

Having described the ROC curves, the first element of further analysis is to calculate the corresponding PROC curves for each of the four scenarios. The required probabilities can be obtained by adopting a value of *p_c_* (prevalence), systematically varying the position of the threshold on the risk score scale to obtain values of *p*_+|*c*_ (TPP) and *p*_+|*nc*_ (FPP = 1 − TNP), then calculating PPV and 1 − NPV via Bayes’ Rule. For each scenario, a PROC curve is calculated for each of nine prevalence values, from *p_c_* = 0.1 to 0.9 at intervals of 0.1 (Figure 2, Figure 3, Figure 4 and Figure 5).

As noted in [2], the shapes of PROC curves can appear rather complicated. There is not, as yet, an accepted vocabulary for discussion of the shapes of PROC curves. Here, we offer a descriptive account, based on [2,4]. The PROC curves in Figure 3 and Figure 4, corresponding to ROC curves in Figure 1B (Scenario 2) and Figure 1C (Scenario 3) respectively, do not cross the main diagonal of the PROC plot. Since we know from [4] that where a PROC crosses the diagonal, it does so at the same risk score threshold as the corresponding ROC curve, this suggests that neither ROC curve crosses the diagonal. We know this definitively to be the case for Scenario 2, based on a proper ROC curve.

The PROC curves in Figure 2 and Figure 5, corresponding to ROC curves in Figure 1A (Scenario 1) and Figure 1D (Scenario 4) respectively, cross the main diagonal of the PROC plot. Qualitatively, the shape of these PROC curves resembles that of Figure 2B in [4]. Starting at the left-hand vertical (PPV) axis of the plot, the risk score threshold increases along the curve. The curve cuts the main diagonal of the plot from above, then continues until meeting the horizontal (1 − NPV) axis. Now consider the ROC curves in Figure 1A (for corresponding PROC curves in Figure 2) and Figure 1D (for corresponding PROC curves in Figure 5). From [4], we know that these ROC curves must also cross the diagonal (in fact, they must cross at the same risk score threshold as the corresponding PROC curve). Starting in the top right-hand corner of the ROC plot (FPP = 1, TPP = 1), the risk score threshold increases along the curve. The curve cuts the main diagonal of the plot from above, then continues to the bottom left-hand corner of the plot (FPP = 0, TPP = 0). The point where the ROC curve cuts the diagonal is close to the bottom left-hand corner of the plot in Figure 1A,D, so is not obvious from visual inspection.

At the point where an ROC curve cuts the main diagonal of the plot, TPP = 1 − FPP, and we know that the positive and negative likelihood ratios (LR+ and LR−, respectively) are both equal to 1. Now, via the odds form of Bayes’ Rule (i.e., posterior odds = prior odds × LR(+ or − as appropriate)), the posterior odds of *c* (given either a + or − test result) is equal to the prior odds of *c*; and similarly the posterior odds of *nc* (given either a + or − test result) is equal to the prior odds of *nc*. Converting these odds back to probabilities, we have *p_c_*_|+_ = *p_c_*_|−_ = *p_c_* and *p_nc_*_|+_ = *p_nc_*_|−_ = *p_nc_*. In words, the result means that application of a test based on a threshold positioned on the main diagonal of an ROC plot is uninformative because it results in no revision of prior probabilities to new posteriors. This is a well-known observation; we include it here in order to compare the corresponding observation for a PROC curve. The points where the corresponding PROC curves cut their respective diagonals are (Figure 2 and Figure 5) visually much clearer. We note that when the PROC curve crosses the diagonal of the plot, it does so at the point (1 − NPV, PPV), where both these conditional (posterior) probabilities are equal to the prior, *p_c_*. So we can see directly that a test based on a threshold positioned on the main diagonal of an PROC plot is, by definition, uninformative.

### 2.3. Performance Measures for ROC Curves and Corresponding PROC Curves

Performance measures for ROC and PROC curves are metrics that summarize the consequences of different choices about the position of the threshold on the risk score scale. Thus they provide methods for identification of what Reibnegger and Schrabmair [3] called the “optimum binary cut-off threshold”. In [3] three such methods for ROC curves are considered in a simulation study: a probability-scale metric, an information-scale metric, and a metric based on logistic regression. Here we consider further the first two of these, but do not pursue their logistic regression analysis.

For ROC curves, Reibnegger and Schrabmair [3] calculated the probability-scale metric Youden’s index [12], where the index J = TPP + TNP − 1 = TPP − FPP. J was originally proposed as a generic index for rating diagnostic tests, without reference to ROC curves. For a geometrical interpretation of J in the context of a test with TPP and FPP described by an ROC curve, consider two points on the ROC plot. The first is a point on the ROC curve positioned at a value TPP on the vertical axis; the second a point vertically below the first, positioned on the main diagonal of the plot (where TPP = FPP). The vertical distance between the two points is thus TPP − FPP. J can thus be thought of as the vertical distance between the curve and the main diagonal on an ROC plot at a given value of TPP. Reibnegger and Schrabmair sought the optimum risk score threshold on an ROC curve by systematically varying the threshold and observing the value at which J was maximized. In practice, a search for the maximum value of J would only need to consider thresholds where the ROC curve was above the main diagonal of the plot.

**Figure 6:**
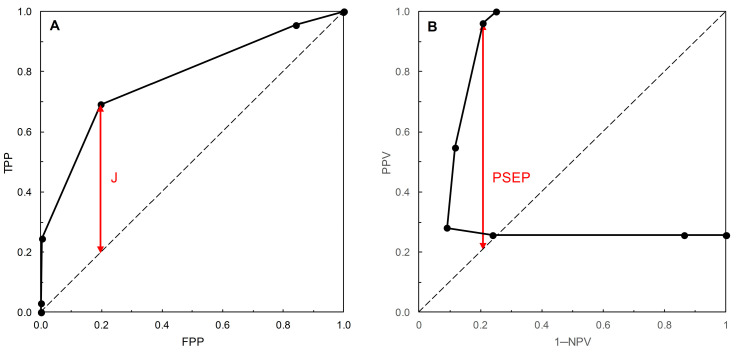
(**A**) The ROC curve is based on the normal distribution, with *c* subjects being N~(1.72, 0.42) and *nc* subjects N~(1.27, 0.27) (see [1] for details). Qualitatively, the shape of this improper ROC curve resembles that of Figure 1C in [4]. The risk score threshold increases along the ROC curve from the top right-hand corner of the plot to the bottom left-hand corner, crossing the main diagonal from below close to the top right-hand corner. The approximate maximum value of J = 0.494 (correct to 3 d.p.) occurs at a risk score threshold of 1.5. (**B**) The corresponding PROC curve was calculated as outlined in [4], with prevalence set to 180/702 = 0.256 (see [1]). Qualitatively, the shape of this PROC curve resembles that of Figure 2C in [4]. The risk score threshold increases along the PROC curve from the right-hand upright of the plot (where 1 − NPV = 1) to the upper horizontal (where PPV = 1), crossing the main diagonal from below at 1 − NPV = PPV = 0.256 (prevalence). The approximate maximum value of PSEP = 0.754 (correct to 3 d.p.) occurs at a risk score threshold of 2.0. Risk score thresholds on both curves are calibrated in units of 0.5 on a −10 to +10 scale (resulting data points may overlap).

Now consider the equivalent geometrical examination of two points on a PROC plot. The first point is on the PROC curve positioned at a given value of PPV on the vertical axis (and, in practice, above the main diagonal of the plot); the second is a point vertically below the first, positioned on the main diagonal of the plot (where PPV = 1 − NPV). The vertical distance between the two points is thus calculated as PPV − (1 − NPV) = PPV + NPV − 1. This probability-scale metric was discussed in the context of the evaluation of diagnostic tests by Altman and Royston [13], who referred to it as PSEP. Note that Altman and Royston’s discussion was generic. It concerned neither ROC curves nor PROC curves. In the present context, one could seek the optimum risk score threshold on an PROC curve by systematically varying the threshold and observing the value at which PSEP was maximized. These geometrical interpretations of the performance measures J (as applied to ROC curves) and PSEP (as applied to PROC curves) are both illustrated in Figure 6. The maximum values of J and of PSEP occur at different risk score thresholds.

We note that the metric *r* = (1 − PPV) + (1 − NPV) = 1 − PSEP [4] was discussed as a performance measure for PROC curves by Shiu and Gatsonis [2] (without reference to PSEP). It is a measure of distance (but not the shortest distance) between a given point on a PROC curve and the point (0, 1) in the top left-hand corner of the plot, with minimum value denoted r*. In passing, we note that the ROC curve analogue of *r* is 1 − J = (1 − TPP) + (1 − TNP). We did not find any discussion of the use of this metric as a performance measure in the literature. The distance metrics J (and its complement) (for ROC curves) and PSEP and *r* (for PROC curves), and other metrics derived from them, have application in graphical determination of thresholds, as discussed in, for example, [1] (see Strategies 5 and 6) and [14].

We turn now to the information-scale metric mutual information (denoted here *I*). In the present context, mutual information is the expected value of the amount of information gained by application of a diagnostic test. Metz et al. [15] and McNeil et al. [16] appear to have described the first applications of *I* in the particular context of ROC curve analysis. As with J and PSEP, *I* is not defined specifically for such application [17]. Reibnegger and Schrabmair [3] sought the optimum risk score threshold on an ROC curve by systematically varying the threshold and observing the value at which *I* was maximized. Here we extend this approach to include the study of both ROC and PROC curves. Hughes [4] briefly discussed *I* as a potential performance measure for PROC curves.

Starting from a generic 2 × 2 prediction-realization table (Table 2), and working in natural logarithms, we obtain mutual information *I* via:

(1) $$I=\sum_{i=+,-}\;\sum_{j=c,nc}p_{i\cap j}\cdot \ln\left\{\frac{p_{i\cap j}}{p_{i}\cdot p_{j}}\right\}$$

from which, on substituting the appropriate numerical data, we may calculate the required estimates of *I* in nats. In the present study, the calculation of *I* via Equation (1) was carried out on systematically varying the risk score threshold over the range 1 to 30 (in increments of 1 unit, along the calculated ROC curves for each scenario shown in Figure 1). In order to apply the results to the corresponding PROC curves (Figure 2, Figure 3, Figure 4 and Figure 5), these calculations were carried out using nine different prior probabilities (prevalence values) over the range 0.1–0.9 in increments of 0.1.

We note at this stage that Equation (1) can be viewed either from an ROC curve perspective (i.e., in terms of sensitivity and specificity and their complements) or from a PROC curve perspective (i.e., in terms of predictive values). For the ROC perspective, we rewrite Equation (1) as:

(2) $$\begin{array}{cc} I & =p_{+|c}\cdot p_{c}\cdot \ln\left\{\frac{p_{+|c}}{p_{+|c}\cdot p_{c}+p_{+|nc}\cdot p_{nc}}\right\}\\ & +p_{+|nc}\cdot p_{nc}\cdot \ln\left\{\frac{p_{+|nc}}{p_{+|c}\cdot p_{c}+p_{+|nc}\cdot p_{nc}}\right\}\\ & +p_{-|c}\cdot p_{c}\cdot \ln\left\{\frac{p_{-|c}}{p_{-|c}\cdot p_{c}+p_{-|nc}\cdot p_{nc}}\right\}\\ & +p_{-|nc}\cdot p_{nc}\cdot \ln\left\{\frac{p_{-|nc}}{p_{-|c}\cdot p_{c}+p_{-|nc}\cdot p_{nc}}\right\} \end{array}$$

in nats, which is Equation (2) from [15] written in the notation of the current article. Here mutual information is written as a function of sensitivity and specificity (and their complements) and the prevalence values for cases and non-cases. For the PROC perspective, we rewrite Equation (1) as:

(3) $$I=\sum_{i=+,-}p_{i}\sum_{j=c,nc}p_{j|i}\cdot \ln\left\{\frac{p_{j|i}}{p_{j}}\right\}$$

in nats, which is Equation (4) from [18] written in the current notation. Here, mutual information is written as the information obtained from a specific test outcome (either + or −) averaged over both *c* and *nc* subjects (this is relative entropy), then averaged over both + and − outcomes. Both [15] and [18] worked in base 2 logarithms rather than natural logarithms. To convert from natural logarithms to base 2 logarithms, divide by ln(2) ≈ 0.693 (in which case the units are bits).

## 3. Results

An immediate consequence of the fact that Equation (1) can be viewed either from the perspective of an ROC curve (Equation (2)) or a PROC curve (Equation (3)) is that the mutual information calculated for a given 2 × 2 prediction-realization table applies to the same risk score threshold on both curves. Thus, mutual information as a performance measure for binary predictors characterized by both ROC and PROC analysis has the same value at the same risk score threshold on both curves. Having obtained this result, we do not pursue the separate probability metrics J (for ROC curves) and PSEP (for PROC curves) further. We focus instead on the information metric *I*, applicable to both curves.

It is tests based on the part of the ROC curve above the main diagonal of the plot that are of interest in the context of diagnostic decision making. Here, *p*_+|*c*_ > *p*_+|*nc*_, which implies *p_c_*_|+_ > *p_c_* and *p_nc_*_|−_ > *p_nc_* [4]. And as noted above, we know from [4] that for an ROC curve that crosses the main diagonal of the ROC plot, the corresponding PROC curve crosses the main diagonal of the PROC plot at the same threshold risk score. Looking first at Equation (2), recall that *p_c_* + *p_nc_* = 1, and that at the point where the ROC curve crosses the diagonal, *p*_+|*c*_ = *p*_+|*nc*_ and *p*_−|*c*_ = *p*_−|*nc*_. Thus at that point, each of the four terms in curly brackets in Equation (2) is equal to 1, and as ln{1} = 0, *I* = 0 nats. Looking now at Equation (3), recall that where the PROC curve crosses the diagonal of the plot, we have *p_c_*_|+_ = *p_c_*_|−_ = *p_c_* and *p_nc_*_|+_ = *p_nc_*_|−_ = *p_nc_*. So in Equation (3), we again have four terms in curly brackets, each term equal to 1 at the point where the PROC curve crosses the diagonal, so again we have *I* = 0 nats. This result confirms that at the risk score threshold where an ROC curve and the corresponding PROC curve cross the main diagonal of their respective plots, characterizing an uninformative predictor, the mutual information *I* is zero nats.

**Figure 7:**
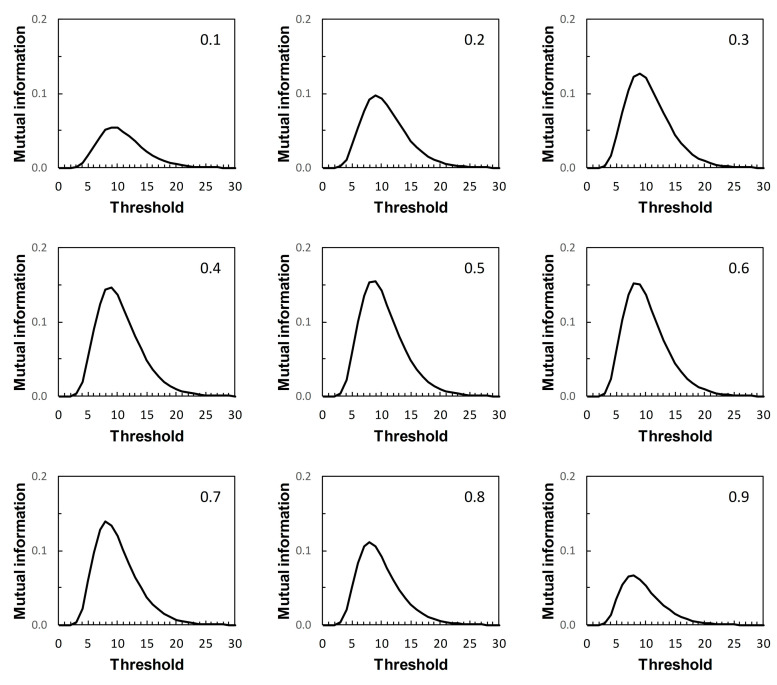
Scenario 1: variation of mutual information with risk score threshold. The calculated values of mutual information apply at risk score thresholds on the ROC curve in Figure 1A and at the same risk score thresholds on the corresponding PROC curves in Figure 2. Each panel is labelled with the prevalence value at which the graph was calculated. The vertical axis scales on Figure 7, Figure 8, Figure 9 and Figure 10 differ.

**Figure 8:**
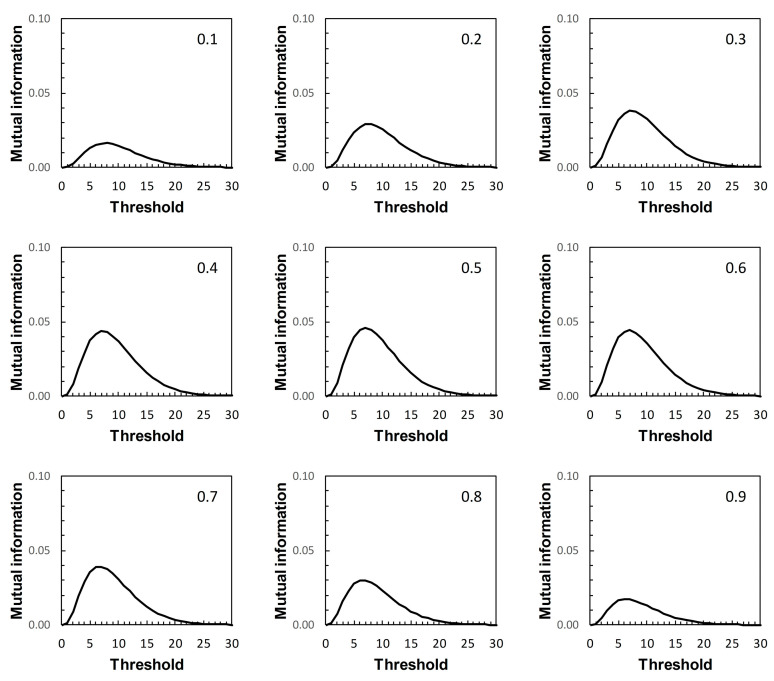
Scenario 2: variation of mutual information with risk score threshold. The calculated values of mutual information apply at risk score thresholds on the ROC curve in Figure 1B and at the same risk score thresholds on the corresponding PROC curves in Figure 3. Each panel is labelled with the prevalence value at which the graph was calculated. The vertical axis scales on Figure 7, Figure 8, Figure 9 and Figure 10 differ.

**Figure 9:**
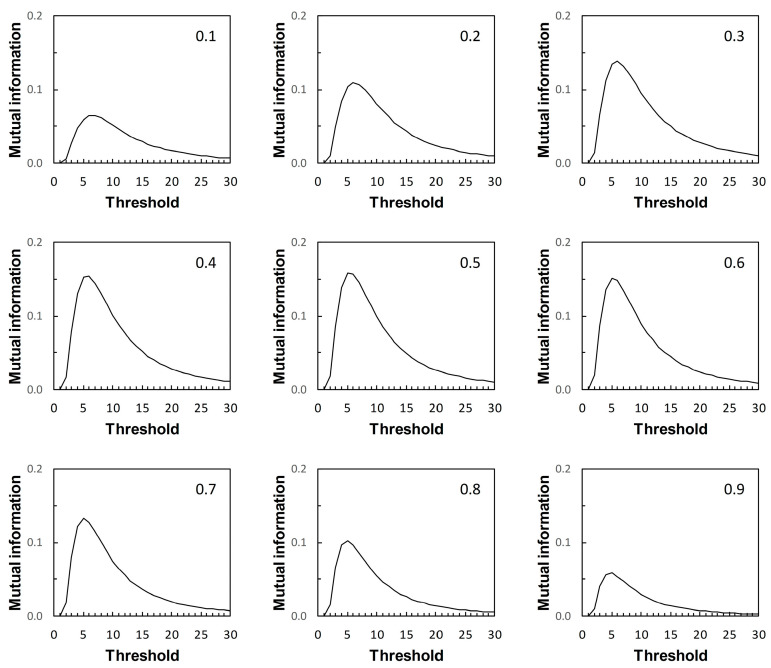
Scenario 3: variation of mutual information with risk score threshold. The calculated values of mutual information apply at risk score thresholds on the ROC curve in Figure 1C and at the same risk score thresholds on the corresponding PROC curves in Figure 4. Each panel is labelled with the prevalence value at which the graph was calculated. The vertical axis scales on Figure 7, Figure 8, Figure 9 and Figure 10 differ.

**Figure 10:**
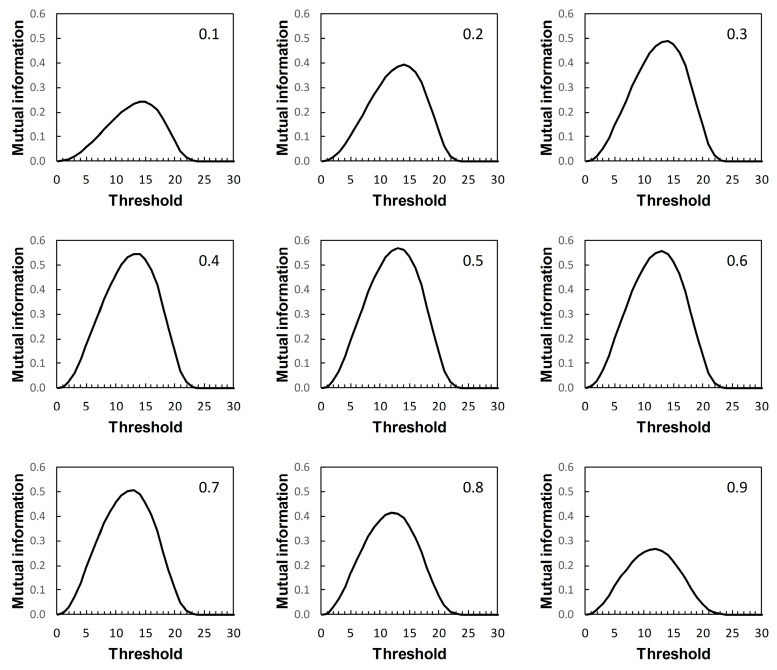
Scenario 4: variation of mutual information with risk score threshold. The calculated values of mutual information apply at risk score thresholds on the ROC curve in Figure 1D and at the same risk score thresholds on the corresponding PROC curves in Figure 5. Each panel is labelled with the prevalence value at which the graph was calculated. The vertical axis scales on Figure 7, Figure 8, Figure 9 and Figure 10 differ.

We now return to the scenarios outlined in Table 1. These are arbitrary in the sense that they represent plausible statistical simulacra of data used in the context of diagnostic test evaluation, rather than any specific disease diagnostic scenario. So the results presented here (Figure 7, Figure 8, Figure 9 and Figure 10) are of interest mainly in terms of their qualitative characteristics. Note, in particular, that in the examples presented there is always a single maximum value of *I* (referred to here as *I_max_*) over the range of threshold risk scores, whatever the shapes of the ROC and PROC curves. Somoza and Mossman [19] also observed this in a study based on bi-normal ROC curves. The threshold risk score for *I_max_* decreases slowly with increasing prior probability, as noted in Reibnegger and Schrabmair’s simulation study [3].

For each of Figure 7, Figure 8, Figure 9 and Figure 10, each of the nine panels shows how *I* varies with risk score threshold at a specified prior probability. *I_max_* refers to the maximum value of *I* for a particular panel. Clearly there is variation in *I_max_* over the set of panels in each of Figure 7, Figure 8, Figure 9 and Figure 10. Recall that in Figure 7, Figure 8, Figure 9 and Figure 10, each panel applies both to an ROC curve from Figure 1A–D respectively and to a PROC curve from the corresponding panel from Figure 2, Figure 3, Figure 4 and Figure 5 respectively. The values of *I_max_* obtained in this way characterize an information-scale specification of the optimum risk score threshold at a specified prevalence for an ROC curve as discussed by [3], which is shown here to apply also to the corresponding PROC curves.

Metz et al. [15] were not directly concerned with characterizing the optimum risk score threshold on an ROC curve. Instead, their application of *I_max_* was as measure of the “system quality” attributable to a device used in diagnostic decision making and described by an ROC curve, for the purpose of comparison with other such devices. Nevertheless, the calculations of mutual information in [15] are the same as those required for application in characterizing ROC curve thresholds [3], and those presented here with application further extended to characterizing PROC curve thresholds.

Metz et al. [15] pointed out a distinction between *I_max_* and the global “information capacity” of a system. Information capacity, which we refer to here as channel capacity (denoted *C*) is the maximum value of *I* at a given risk score threshold taken over all values of prevalence. A (binary) “channel”, in this case, is represented quantitatively by data from a numerical version of a 2 × 2 table such as Table 2. Now, for example, suppose we obtain from Figure 7, Figure 8, Figure 9 and Figure 10 the risk score thresholds at which the largest value of *I_max_* is observed for each scenario. These thresholds occur at 9 (Scenario 1, Figure 7), 7 (Scenario 2, Figure 8), 5 (Scenario 3, Figure 9), and 13 (Scenario 4, Figure 10). The corresponding largest observed values of *I_max_* for each respective specified risk score threshold are then *I_max_* = 0.154 nats (Figure 7), *I_max_* = 0.046 nats (Figure 8), *I_max_* = 0.158 nats (Figure 9) and *I_max_* = 0.568 nats (Figure 10). We note in passing that these values of *I_max_* reflect our earlier visual description of the ROC curves for the four scenarios in terms of the relative proximity of their paths to the top left-hand corner of the plot (Figure 1).

What we cannot say without further analysis is that these estimates of *I_max_* are in the vicinity of *C*. While the calculation of *C* from a general prediction-realization table requires application of an iterative algorithm, there is a relatively simple analytical solution available in the case of a channel represented by a 2 × 2 table [20,21]. From this, using the same thresholds as above, we obtain for Scenario 1, *C* = 0.155 nats; for Scenario 2, *C* = 0.046 nats; for Scenario 3, *C* = 0.158 nats; and for Scenario 4, *C* = 0.569 nats (all to 3 d.p.). We find that the maximum value of *I_max_*, obtained graphically at specified thresholds from Figure 7, Figure 8, Figure 9 and Figure 10 for each of the four scenarios, is an approximation of the corresponding value of *C*. Thus calculation of the maximum value of *I_max_* at a specified threshold can provide an estimate of what Metz et al. [15] called information capacity, furnishing an upper limit to their information theoretic measure of system quality. This result was unforeseen by Metz et al. [15].

## 4. Discussion

Vermont et al. [1], concluding their study of the roles of ROC curves and PROC curves in the context of graphical methods for diagnostic threshold determination, wrote as follows: “we do not feel that it is possible to choose a segmentation threshold by only using the ROC curve of a variable when this threshold must be used for diagnostic purposes; the PROC curves are less attractive, more chaotic and imprecise than the ROC curves but can help to select or reject certain threshold choice strategies”. Much the same point—that the complex patterns of PROC curves made their implementation difficult—was later made by Shiu and Gatsonis [2]. The question thus arises as to how we may realize the advantages of PROC curves in application (that is to say, how to make them more attractive) in the face of apparent presentational difficulties. Answering this question would facilitate use of PROC curve analysis to augment what we can learn from the application of ROC curve analysis, not to substitute for it.

Because of the dependence of PROC curves on prevalence, we displayed an array of PROC curves corresponding to the ROC curve on which each scenario was based (Figure 2, Figure 3, Figure 4 and Figure 5). When calibrating predictive values for a predictor initially based on an ROC curve, there is potential application for an array of PROC curves such as shown in each of Figure 2, Figure 3, Figure 4 and Figure 5 if consideration of more than one prevalence value is deemed necessary. For example, it was noted in [22] that the prevalence of bladder cancer is known to differ between subgroups of males and females. In such a situation, an array of PROC curves for different prevalence values may allow a preview of the likely extent of differences between the curves for each of the subgroups. A similar situation may arise in crop protection decision making with a predictor based on an ROC curve. For example, a predictor may be used in separate locations where geographical and/or climatic differences result in subgroups with differing disease prevalence [23].

Vermont et al. [1] discussed strategies for threshold determination based on probability measures; sensitivity and specificity for ROC curves, predictive values for PROC curves. We have discussed examples of such measures; J [12] for ROC curves and its analogue PSEP [13] for PROC curves. Probability measures require separate calculation and interpretation of performance measures for ROC curve analysis and for PROC curve analysis. Mutual information is an information theoretic performance measure that has had application in the analysis of ROC curves, for example [3,15,16]. We have studied the concurrent application of mutual information to the analysis of ROC curves and their corresponding PROC curves. The important new result of our study is that mutual information is a performance measure that is applicable to the analysis of both ROC curves and PROC curves. In particular, for a given prevalence, mutual information calculated at a specified risk score threshold on an ROC curve (using Equation (2)) is the same as mutual information calculated at the same risk score threshold on a PROC curve (using Equation (3)). In our study this result applied to scenarios based on proper, improper, and unspecified-type ROC curves. It is also applicable to empirical ROC and PROC analysis, as for example in [22].

The presentation of this result is noteworthy. We begin with an ROC curve, the graphical plot of sensitivity (TPP) against 1 − specificity (1 − TNP = FPP) (e.g., Figure 1). This curve is independent of prevalence. However, a PROC curve, the graphical plot of positive predictive value (PPV) against 1 − negative predictive value (1 − NPV), is not independent of prevalence. Thus, in our study, we calculate PROC curves corresponding to an ROC curve for a range of prevalence values, from 0.1 to 0.9 at intervals of 0.1. Then, in each of Figure 2, Figure 3, Figure 4 and Figure 5, we present an array of nine PROC curves for each ROC curve shown in Figure 1. Now we can calculate mutual information for risk score thresholds from 1 to 30 at intervals of 1 unit (thus following the methodology of [3]). These mutual information values apply to risk score thresholds along the ROC curve and to the same thresholds along the corresponding array of PROC curves. Thus, if we describe a scenario for description of a diagnostic device in terms of an ROC curve and a set of likely prevalence values in which the device may be operational, we can present an array of graphical plots of mutual information against risk score threshold as a performance measure that applies both to the ROC curve and the corresponding PROC curves (e.g., Figure 7, Figure 8, Figure 9 and Figure 10).

If we set out to integrate ROC curve analysis and PROC curve analysis into a strategy for graphical threshold determination [1], an array such as shown in each of Figure 7, Figure 8, Figure 9 and Figure 10 provides an information theoretic basis on which to meet this objective. We note that the threshold at which *I_max_* is indicated in the appropriate panel of an array (for the specified prevalence) is not prescriptive. It provides guidance towards the choice of an appropriate threshold, taking into consideration data on both sensitivity and specificity (via the ROC curve) and predictive values (via the PROC curve). Values of sensitivity, specificity (and so J) and predictive values (and so PSEP) in the vicinity of the threshold identified by *I_max_* can always be investigated if required.

Drawing mutual information contours calculated at a specified prevalence onto ROC space [15] is another way in which to present the information theoretic analysis of an ROC curve. However, this approach does not allow for integration of an analysis of the corresponding PROC curves into the same graphic. Nor, we believe, does this contour plot depict *I_max_* as clearly as a graph of mutual information against risk score threshold. Metz et al. [15] were concerned with measuring and comparing system quality via mutual information, specifically by calculating *I_max_* from an ROC curve by means of Equation (2) applied at a given prevalence. Any one panel from an array of graphical plots of mutual information against risk score threshold (e.g., Figure 7, Figure 8, Figure 9 and Figure 10) fulfils this objective for a particular prevalence value. In addition, the maximum value of *I_max_* at a specified risk score threshold across an array, independent of prevalence, is an estimate of channel capacity *C*.

There is little doubt that the complexity of PROC curves [1,2] is an obstacle to their application in assessment of the performance of binary predictors. Equally, few would disagree that predictive values, alongside sensitivity and specificity, should have a role to play in characterizing predictor performance. We have shown that adoption of an information theoretic performance measure, mutual information, in a graphical format that plots the variation of mutual information over an appropriate range of risk score thresholds, allows integration of ROC curve analysis and PROC curve analysis. So the undoubted difficulties of interpretation that the PROC graph’s complexity presents may be avoided, while retaining the benefits of considering predictive values alongside ROC curve characteristics in the evaluation of predictor performance.

## Figures and Tables

**Table 1:** The four analytical scenarios ^i,ii^.

Scenario	Distribution of *c*	Distribution of *nc*
1 ^iii^	Lognormal; mean = 2.5, s.d. = 0.3	Lognormal; mean = 2.0, s.d. = 0.4
2 ^iv^	Chi-squared; d.f. = 10	Chi-squared; d.f. = 7
3	Inverse gamma; shape = 3	Inverse gamma; shape = 6
4	Weibull; shape = 10, scale = 20	Chi-squared; d.f. = 6

^i^ Notation: *c*, cases; *nc*, non-cases; s.d., standard deviation; d.f., degrees of freedom. ^ii^ See Figure 1 in [3] for a graphical illustration of these scenarios. Each distribution was plotted over the range from 1 to 30 on the horizontal axis. ^iii^ See [9] for further discussion of the bi-lognormal receiver operating characteristic (ROC) curve. ^iv^ See [10] for further discussion of the bi-chi-squared ROC curve.

**Table 2:** The prediction-realization table for a test with two categories of realized (actual) status (*c*, *nc*) and two categories of prediction (+, −). In the body of the table are the joint probabilities.

Prediction (*i*)	Realization (*j*)		
	*c*	*nc*	Row Sums
+	*p*_+∩*c*_	*p*_+∩*nc*_	*p*_+_
−	*p*_−∩*c*_	*p*_−∩*nc*_	*p*_−_
Column Sums	*p* *_c_*	*p* *_nc_*	1
